# Do prognosis and clinicopathological features differ in young early-stage breast cancer?

**DOI:** 10.3389/fsurg.2022.900363

**Published:** 2022-10-19

**Authors:** Akif Enes Arikan, Halil Kara, Onur Dülgeroğlu, Esin Nur Erdoğan, Emir Capkinoglu, Cihan Uras

**Affiliations:** ^1^ Department of General Surgery, Acibadem Mehmet Ali Aydinlar University, School of Medicine, Istanbul, Turkey; ^2^ Research Institute of Senology, Acibadem Mehmet Ali Aydinlar University, Istanbul, Turkey; ^3^ Vocational School of Health Sciences, Acibadem Mehmet Ali Aydinlar University, Istanbul, Turkey; ^4^ School of Medicine, Acibadem Mehmet Ali Aydinlar University, Istanbul, Turkey

**Keywords:** age factors, breast cancer, cancer-specific survival, disease-free survival, early stage

## Abstract

**Background:**

Breast cancer is the most frequently detected cancer and the leading cause of cancer-related death in women. Although it is mostly seen in older patients, breast cancer affects women aged 24 to >70 years, with poorer prognosis in young patients. Young age remains a controversial topic in the literature. This study aimed to identify subtype differences and the effect of age on early-stage breast cancer outcomes.

**Methods:**

A total of 300 consecutive patients underwent surgery between 2011 and 2015 for early-stage breast cancer. Of these, 248 were eligible for this study and were divided into three groups: group Y (aged ≤35 years), group M (aged >35 and ≤45 years), and group E (aged >45 years). The clinical and pathological features and data related to recurrence, metastasis, and death were recorded.

**Results:**

No statistical differences were found between groups regarding histopathological features except for higher histological grade and Ki-67 levels in group M. Additionally, group Y recorded no progression (recurrence or metastasis) or death. Disease-free survival was 117.8 months (95% CI 111.8–123.8) for group M, which was significantly shorter than that for group E (*p* < 0.001). Additionally, the hazard ratio (HR) for progression from group M to group E was 10.21 with significant difference (*p* = 0.003, 95% CI 2.26–46.08). However, the HR of group Y to group E was 0.04, without significance (*p* = 0.788, 95% CI 0.18–345 × 10^6^). The overall 5-year survival was 100% in group Y, 98.8% in group M, and 99.3% in group E, without significance.

**Conclusion:**

A very young age cannot be considered an independent risk factor for poor prognosis. Rather than age, histological grade and Ki-67 index are more important factors in early-stage breast cancer.

## Introduction

Breast cancer is the most common cancer among women worldwide (1). The incidence of breast cancer in women starts to increase after the age of 24 years and continues to be more common than any other cancer even after the age of 70 years (2). Evidence showed that a large group of women (aged 25–70 years and older) who are at risk for breast cancer are not entirely candidates for the most aggressive types of tumors because tumor characteristics—and thus prognosis—and responses to treatment differ by age at diagnosis (3).

The cut-off value for young age definition depends on which reveals more differences between age groups, and this is one of the most important topics regarding subtype differences according to age (4, 5). Defining the “young patient” as patients who are ≤45 years old and “very young patient” as ≤35 years old is controversial regarding whether this type of classification is necessary (6). Studies have shown that patients who are diagnosed with breast cancer at a younger age tend to have worse outcomes in three main parameters: developing a metastatic condition, recurrence, and death (7). As a result, in the younger age group, the prognosis and overall survival (OS) rates were worse (8). This is arguably a result of their tumor characteristics based on immunohistochemistry properties [estrogen receptor (ER), progesterone receptor (PR), human epidermal growth receptor-2 (HER-2), and Ki-67 proliferation index] (9–11). According to these phenotypic features, four main subtypes are defined by the American Joint Committee of Cancer (AJCC) as “basal-like (a.k.a. triple-negative),” luminal subtypes (“Luminal A” and “Luminal B”), HER-2 subtype (i.e., HER-2-like or HER-2 enriched) (12).

Evidence suggests that young women also present with more advanced-stage cancer due to their high mitotic index and higher proportion of ER/PR-negative and HER-2 enriched tumors, which results in worse outcomes (11, 13, 14).

Thus, determining whether a major difference exists between age groups in terms of receptor subtypes is an important factor in ruling the treatment and predicting prognosis. Tumor characteristics are central to the diagnosis and treatment of breast cancer. Additionally, differences between age groups may eventually indicate which features of the tumor are worthy of attention, especially in patients aged 45 years and younger (15, 16).

This study aimed to determine whether there are any subtype differences and the effect of age on early-stage breast cancer outcomes.

## Materials and methods

To differentiate tumor subtypes among age groups of women, 300 consecutive patients who had undergone surgery at the affiliated hospitals of Acibadem Mehmet Ali Aydinlar University, Research Institute of Senology (RISA) for primary early-stage breast cancer and did not receive any neoadjuvant treatment were assessed. Patient information regarding tumor characteristics and follow-up data were extracted from the RISA database and hospital information system (HIS).

### Inclusion criteria

• Surgery for primary, nonmetastatic, early-stage (i.e., stages I–II according to the pathology report) breast cancer,
• Underwent surgery as first-line treatment,
• Tumor category was pT1–pT2 according to AJCC Breast Cancer TNM Staging System edition 8 (12), and
• Being operated in the affiliated hospitals of RISA between 2011 and 2015.

### Exclusion criteria

• Staged as III or IV,
• Received neoadjuvant chemotherapy, and
• Metastasis either by radiologic, metabolic, or pathological diagnosis.

Patients were grouped according to their age into group Y (aged ≤35 year), group M (aged >35 and ≤45 years), and group E (aged >45 years). Tumor characteristics were noted according to the latest guidelines of the AJCC on Cancer Staging and Biological Classification (12). To determine biological features, hormone receptors were investigated with immunohistochemical staining, and Her2 was further assessed by fluorescence *in situ* hybridization if it was equivocal. Her2 was noted as negative or positive. Furthermore, the tumor biology was classified as luminal A-like, luminal B-like, HER2 overexpression, and basal-like (17). The AJCC defines early-stage breast cancer as stages I–II (12).

After the operation, adjuvant treatment was determined by a multidisciplinary tumor board for each patient according to the current guidelines. Follow-up data for metastasis and recurrence were collected from the HIS. Metastasis, recurrence, and death were considered indicators of disease outcome and prognosis at follow-up. The date of the last information was recorded according to the date of the last physical examination, imaging report, or laboratory test results in the HIS. Clinical node positivity was assessed by clinical examination, radiological assessment, or 18-fluorodeoxyglucose positron-emission tomography/computed tomography reports.

### Statistics

All analyses were performed using IBM SPSS Statistics for Windows, Version 25.0 (IBM Corp., Armonk, NY, United States). Descriptive analyses, chi-square test, Fisher’s exact test, one-way analysis of variance test, Welch’s *F* test, Tukey’s honestly significant difference (HSD) *post-hoc* test, Student’s *t*-test, Kaplan–Meier test, and Cox regression analysis were performed. Three-group (group Y vs. group M vs. group E), two-group (group Y + M vs. group E), or in-group (group Y vs. group M) comparisons were performed. Statistical significance was defined as a *p* < 0.05, and the confidence interval was calculated as 95% (95%CI).

## Results

In total, of 300 consecutive patients with early-stage breast cancer, 19 were excluded because of previous breast cancer recurrence or missing major information. Additionally, 33 patients (2 were ≤35 years old, 23 were >45 years old, and 8 were in between) had stage III or IV according to the pathology report; thus, they were excluded.

Of the 248 included patients, 14 were in group Y, 83 were in group M, and 151 were in group E. Pregnancy-related breast cancer was not observed in this study. The demographic and clinical features are shown in Table 1. The type of operations performed and status of adjuvant treatments are given in Table 2.

**Table 1:** Demographic and clinical features.

	Group Y (*n* = 14)	Group M (*n* = 83)	Group E (*n* = 151)	Overall (*n* = 248)	*p*-value
Age (mean ± SD) (min–max)	33.2 ± 2.9 (25–35)	41.5 ± 2.9 (36–45)	55 ± 6.7 (46–77)	49.3 ± 9.3 (25–77)	
History of breast cancer in first degree relative (s)	1 (7%)	5 (6%)	9 (6%)	15 (6%)	0.91^a^
Has complaint					
No	2 (14.3%)	19 (22.9%)	45 (29.8%)	66 (26.6%)	0.33^a^
Yes	12 (85.7%)	64 (77.1%)	106 (70.2%)	182 (73.4%)	
Burning, pain, tingling	—	1	1	2	1^a^
Mass, stiffness, swelling	14	70	124	208	0.78^a^
Nipple discharge	—	2	—	2	0.25^a^
Nipple/skin retraction	1	3	4	8	0.31^a^
Side of the tumor					
Left	6 (42.9%)	47 (56.6%)	61 (41.8%)	114 (46.9%)	0.09
Right	8 (57.1%)	36 (43.4%)	85 (58.2%)	129 (53.1%)	
Bilateral	—	—	5	5	0.29^a^
Has palpable tumor					
No	1 (7%)	17 (20.5%)	36 (24%)	54 (22%)	0.37^a^
Yes	13 (93%)	66 (79.5%)	115 (76%)	194 (78%)	
Clinical tumor size (mm) (mean ± SD) (min–max)	20 ± 5 (15–30)	21 ± 9 (6–50)	20 ± 8 (3–50)	21 ± 8 (3–50)	0.63
Clinical tumor category					
cT1	9 (64%)	60 (72%)	105 (79.5%)	174 (70%)	0.76^a^
cT2	5 (36%)	23 (28%)	46 (30.5%)	74 (30%)	
Clinical lymph node category					
cN0	11 (78.6%)	61 (73.5%)	122 (80.8%)	194 (78.2%)	0.45^a^
cN1	3 (21.4%)	22 (26.5%)	29 (19.2%)	54 (21.8%)	
Clinical anatomic staging					
IA	7 (50%)	45 (54%)	94 (62%)	146 (59%)	0.33^a^
IIA	6 (43%)	31 (37%)	39 (26%)	76 (31%)	
IIB	1 (7%)	7 (8%)	18 (12%)	26 (11%)	

^a^ Fisher’s exact test was utilized.

**Table 2:** Type of surgical and adjuvant treatments according to age groups.

	Group Y (*n* = 14)	Group M (*n* = 83)	Group E (*n* = 151)	Overall (*n* = 248)	*p*-value
Type of performed breast surgery					
Breast conserving surgery	12 (85.7%)	69 (83.1%)	98 (64.9%)	179 (72.2%)	**0.005** ^a^
Mastectomy	2 (14.3%)	14 (16.9%)	53 (35.1%)	69 (27.8%)	
Simple mastectomy	—	2 (14%)	21 (40%)	23 (33%)	0.13^a^
Subcutaneous mastectomy	2 (100%)	12 (86%)	32 (60%)	46 (67%)	
Nipple sparing mastectomy	1 (50%)	10 (83%)	22 (69%)	33 (72%)	0.50^a^
Skin sparing mastectomy	1 (50%)	2 (17%)	10 (31%)	13 (28%)	
Type of performed axillary surgery					
Sentinel lymph node biopsy					
Not performed	0	1^b^		1^b^	
Negative	9 (64%)	51 (61%)	118 (78%)	178 (72%)	**0.025** ^a^
Positive	5 (36%)	31 (39%)	33 (22%)	69 (28%)	
** **Axillary lymph node dissection					
Performed	3 (60%)	26 (84%)	32 (97%)	61 (88.4%)	**0.025** ^a^
Not Performed	2 (40%)	5 (16%)	1 (3%)	8 (11.6%)	
Status of adjuvant chemotherapy^c^					
Received	11 (79%)	62 (75%)	71 (47%)	144 (58%)	**<0.001** ^a^
Not received	2 (14%)	21 (25%)	79 (52%)	102 (41%)	
Unknown	1 (7%)	0 (0%)	1 (1%)	2 (1%)	
Status of adjuvant hormonotherapy^c^					
Received	2 (14%)	11 (13%)	20 (13%)	33 (13%)	0.95^a^
Not received	11 (79%)	72 (87%)	130 (86%)	215 (87%)	
Unknown	1 (7%)	0 (0%)	1 (1%)	4 (2%)	
Status of adjuvant radiotherapy^c^					
Received	13 (93%)	79 (95%)	112 (74%)	204 (82%)	**<0.001** ^a^
Not received	0 (0%)	3 (4%)	37 (25%)	40 (16%)	
Unknown	1 (7%)	1 (1%)	2 (1%)	4 (2%)	

^a^ Fisher’s exact test was utilized.

^b^ Only mammaria interna lymph node was sought surgically, so this patient was excluded from analysis.

^c^ Calculation was performed with exclusion of unknown cases.

Statistically significant p-values were written in bold.

### Tumor characteristics

No significant differences were found in anatomic stages among the three-group (*p* = 0.33), two-group (*p* = 0.11), and in-group (*p* = 0.91) comparisons.

In groups Y and E, in contrast to group M, the histologic grade (HG) was 2 in more than 50% of the patients. This distribution of HG showed significant differences in the three-group (*p* = 0.002) and two-group (*p* = 0.001) comparisons; however, it did not show significant difference in the in-group comparison (*p* = 0.18).

Ki-67 levels were significantly different in the three- and two-group comparisons (*p* < 0.001 for both), with the lowest level in group E and the highest in group M; however, no significant differences were noted in the in-group comparison (*p* = 0.20). Additionally, Ki-67 was grouped into low (0%–9%), medium (10%–19%), and high (>19%), which yielded significant differences in the three- and two-group comparisons (*p* = 0.013 for both), but not in the in-group comparison (*p* = 0.13). Further comparison of the Ki-67 groups between group Y vs. group E did not sustain a significant difference (*p* = 0.33), compared to group M vs. group E (*p* = 0.008). All the histopathological characteristics are shown in Table 3.

**Table 3:** Pathological characteristics of the patients.

	Group Y (*n* = 14)	Group M (*n* = 83)	Group E (*n* = 151)	Overall (*n* = 248)	*p*-value
Pathological tumor size (mm) (mean ± SD) (min–max)	20.5 ± 11.8 (5–45)	18.2 ± 10.1 (4–48)	17.2 ± 8.8 (2–45)	17.7 ± 9.4 (2–48)	0.38
Pathological tumor category					
pT1	9 (64%)	59 (71%)	107 (71%)	175 (71%)	0.87^a^
pT2	5 (36%)	24 (29%)	44 (29%)	73 (29%)	
Pathological lymph node category					
pN0	8 (57%)	52 (63%)	115 (76%)	175 (71%)	0.06^a^
pN1	6 (43%)	30 (37%)	36 (24%)	72 (29%)	
Pathological anatomic staging					
IA	5 (36%)	37 (45%)	93 (61.6%)	135 (55%)	**0.012** ^a^
IIA	7 (50%)	36 (44%)	36 (23.8%)	79 (32%)	
IIB	2 (14%)	9 (11%)	22 (14.6%)	33 (13%)	
Histological grade					
1	—	6 (7.2%)	32 (20.5%)	37 (14.9%)	**0** **.** **002** ^a^
2	10 (71.4%)	36 (43.4%)	77 (51%)	123 (49.6%)	
3	4 (28.6%)	41 (49.4%)	43 (28.5%)	88 (35.5%)	
Estrogen receptor (%)	77 ± 40 (0–100)	75 ± 36 (0–100)	81 ± 35 (0–100)	77 ± 40 (0–100)	0.50
Negative	2 (14%)	11 (13%)	20 (13%)	33 (13%)	1^a^
Positive	12 (86%)	72 (87%)	131 (87%)	215 (87%)	
Progesterone receptor (%)	33 ± 34 (0–90)	46 ± 37 (0–100)	44 ± 41 (0–100)	33 ± 34 (0–90)	0.51
Negative	4 (29%)	19 (23%)	43 (29%)	66 (27%)	0.68^a^
Positive	10 (71%)	64 (77%)	108 (72%)	182 (73%)	
Ki-67 (mean ± SD) (median; min–max)	26 ± 18 (21; 5–59)	34 ± 22 (32; 2–95)	23 ± 18 (20; 0–75)	26 ± 18 (22.5; 5–59)	**<0.001**
Low (0%–9%)	2 (14%)	12 (14%)	43 (29%)	57 (23%)	**0** **.** **013** ^a^
Medium (10%–19%)	5 (36%)	12 (14%)	32 (21%)	49 (20%)	
High (20%–100%)	7 (50%)	59 (71%)	76 (50%)	142 (57%)	
Human epidermal growth factor receptor-2					
Amplified/overexpressed	3 (21%)	18 (22%)	25 (17%)	46 (19%)	0.56
Not-amplified/not expressed	11 (79%)	65 (78%)	126 (83%)	202 (81%)	
Biological subtypes					
Luminal	12 (86%)	72 (87%)	131 (87%)	215 (87%)	1^a^
Luminal-A like	3 (21.4%)	24 (28.9%)	54 (35.8%)	81 (32.7%)	0.79^a^
Luminal-B like	9 (64.3%)	48 (57.8%)	77 (51%)	134 (54%)	
Nonluminal	2 (14%)	11 (13%)	20 (13%)	33 (13%)	
Her2 like		4 (4.8%)	6 (4%)	10 (4%)	
Basal like	2 (14%)	7 (8.4%)	14 (9.3%)	23 (9.3%)	

pT1: Tumor size ≤ 20 mm, pT2: Tumor size > 20 mm and ≤50 mm, pN1: Micrometastases or metastases in 1–3 axillary lymph nodes.

^a^ Fisher’s exact test was utilized.

Statistically significant *p*-values were written in bold.

The difference in tumor biology in terms of “luminal vs. nonluminal,” “luminal-like vs. Her2-like vs. basal-like,” and “luminal-A-like vs. luminal-B-like vs. Her2-like vs. basal-like” for the three-group (*p* = 1, *p* = 0.93, and *p* = 0.79, respectively), two-group (*p* = 0.97, *p* = 1, and *p* = 0.61, respectively), and in-group (*p* = 1, *p* = 0.80, and *p* = 0.79, respectively) comparisons did not reach significance. Additionally, the same biological subtypes were compared between groups Y and E, and no significant difference was observed (*p* = 0.71, *p* = 0.61, and *p* = 0.61, respectively).

### Follow-up data and survival information

The median follow-up for all patients was 91.6 months with a mean value of 93.9 ± 19.2 months. No significant differences were found in terms of average follow-up duration for the three-group (*p* = 0.22), two-group (*p* = 0.22), and in-group (*p* = 0.42) comparisons in addition to the comparison of group Y with group E (*p* = 0.85). However, the median follow-up was slightly longer in group M (96.7 months) than in group E (90.4 months) and group Y (86.6 months) without a significant difference in the three-group (*p* = 0.210), two-group (*p* = 0.19), and in-group (*p* = 0.46) comparisons. Survival data are shown in Table 4.

**Table 4:** Survival data of the patients.

	Group Y (*n* = 14)	Group M (*n* = 83)	Group E (*n* = 151)	Overall (*n* = 248)	*p*-value
Follow-up duration (months) (mean ± SD) (median; min–max)	89.8 ± 17.6 (86.6; 63–129)	96.7 ± 18.6 (96.7; 57–129)	92.7 ± 19.6 (90.4; 5–133)	93.9 ± 19.2 (91.6; 52–133)	0.22
Loco-regional recurrence					
Not observed	14 (100%)	79 (95%)	151 (100%)	244 (98%)	**0.037** ^a^
Observed	—	4 (5%)	—	4 (2%)	
Time to event (months) (mean ± SD) (median; min–max)	—	40.8 ± 11.2 (39; 29–56)	—	40.8 ± 11.2 (29–56)	^b^
Metastasis					
Not observed	14 (100%)	74 (89%)	149 (99%)	237 (96%)	**0.004** ^a^
Observed	—	9 (11%)	2 (1%)	11 (4%)	
Time to event (months) (mean ± SD) (median; min–max)	—	55.3 ± 22.3 (49; 31–89)	57.5 ± 37.5 (57.5; 31–84)	55.7 ± 23.2 (49; 31–89)	0.912^c^
Progression					
Not observed	14 (100%)	72 (87%)	149 (99%)	235 (95%)	**0.001** ^a^
Observed	—	11 (13%)	2 (1%)	13 (5%)	
Time to event (months) (mean ± SD) (median; min–max)	—	51.6 ± 21.9 (39; 29–89)	57.5 ± 37.5 (57.5; 31–84)	52.5 ± 22.8 (39; 29–89)	0.75^c^
Death					
Not observed	14 (100%)	82 (99%)	149 (99%)	245 (99%)	1^a^
Observed	—	1 (1%)	2 (1%)	3 (1%)	
Time to event (months) (mean ± SD) (median; min–max)	—	—^d^	75.5 ± 32.3 (75.5; 52–99)	75.5 ± 32.3 (75.5; 52–99)	–^b^

^a^ Fisher’s exact test was utilized.

^b^ Could not be calculated.

^c^ Student’s t-test was performed.

^d^ No exact time span was known.

Statistically significant *p*-values were written in bold.

Recurrences were observed only in group M (*n* = 4, 5%), which yielded significant differences in the three-group comparison (*p* = 0.037) and two-group comparison (*p* = 0. 023) but not in the in-group comparison (*p* = 1).

Group M had the highest rate of metastasis (11%), while group E had the lowest (1%) (group Y had no metastasis) with a significant difference for the three-group (*p* = 0.004) and two-group (*p* = 0.008) comparisons; however, the in-group comparison did not show a significant difference (*p* = 0.35).

Recurrence and metastasis were grouped together as progression to find disease-free survival (DFS). However, progression was not noted in group Y, the lowest progression ratio (1%, *n* = 2) was in group E, and the highest (13%, *n* = 11) was in group M. The progression ratio yielded a significant difference for the three-group (*p* = 0.001) and two-group (*p* = 0.001) comparisons but not for the in-group comparison (*p* = 0.36).

**Figure 1:**
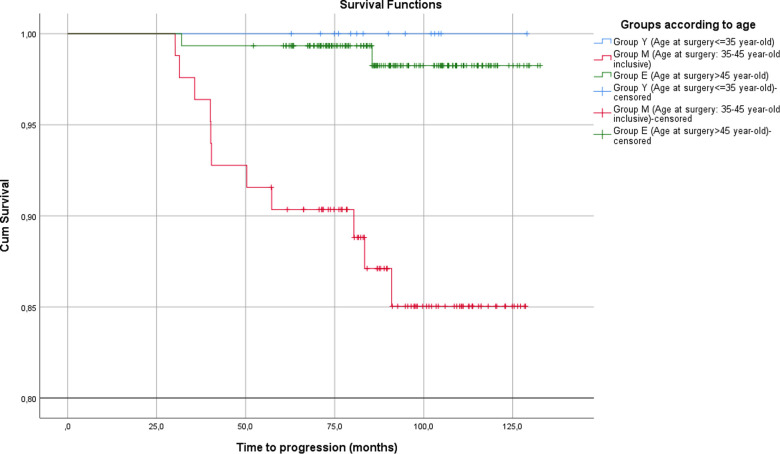
Disease-free survival for each group.

The estimated DFS time in group Y could not be calculated because of a lack of events (Figure 1). DFS was 117.8 months (95%CI 111.8–123.8) for group M, 131.5 (95% CI 129.9–133.2) for group E, and 119.7 (95% CI 114.4–124.9) for groups Y and M. Comparison of the three groups could not be performed; however, significant differences were observed in the two-group comparison (*p* = 0.001) and comparison of group M with group E (*p* < 0.001). The 5-year DFS was 100% in group Y, 90.4% in group M, and 95.34% in group Y.

The hazard ratio (HR) for progression in group Y in comparison with groups E and Y to group M was 0.04 without significance (*p* = 0.788, 95% CI 0.18–345 × 10^6^, *p* = 0.387, 95% CI 0–60.53 respectively). By contrast, the HR of group M to group E was 10.21, showing significant difference (*p* = 0.003, 95% CI 2.26–46.08).

No deaths were observed in group Y, but one (1.2%, related to breast cancer) in group M and two (1.3%; not related to breast cancer) in group E, which did not show any significant difference in the three-, two-, and in-group (*p* = 1 for each) comparisons.

**Figure 2:**
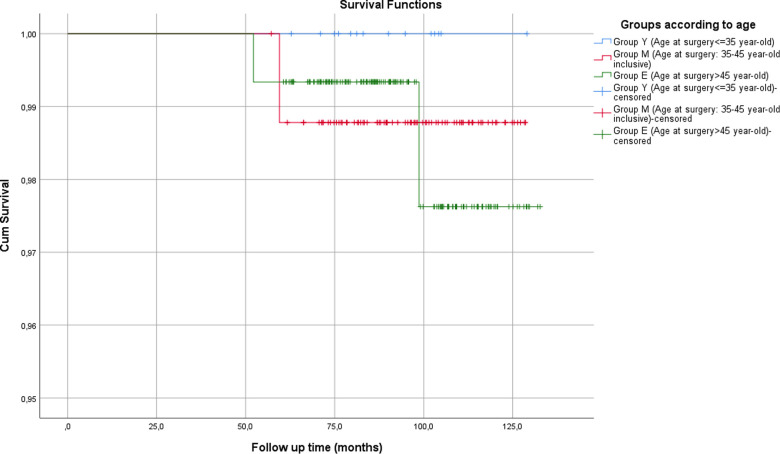
Overall survival for each group.

The 5-year OS was 100%, 98.8%, and 99.3% for groups Y, M, and E, respectively (Figure 2). The estimated OS was 127.8 months (95% CI 126.1–129.4) for group M and 131.6 months (95% CI 130.1–133.1) for group E. The comparison of the OS for two-group (*p* = 0.8) and group M vs. group E (*p* = 0.9) was not significant.

The HR of death for group M to group E was 0.86 (*p* = 0.9, 95% CI 0.08–9.5), for groups Y and M to group E was 0.74 (*p* = 0.81, 95% CI 0.07–8.2), for group Y to group M was 0.04 (*p* = 0.79, 95% CI 0–12 × 10^9^), and for group Y to group E was 0.04 (*p* = 0.78, 95% CI 0–201 × 10^6^); all four were not significantly different.

## Discussion

Some studies have associated young age with a poor prognosis in breast cancer. This study compared clinical features and survival outcomes in early-stage breast cancer among age groups, which showed that being between 35 and 45 years old was associated with a high risk for progression but being ≤35 years old was not.

Diagnosis of advanced-stage cancer in young patients is linked to admission after the development of symptoms (18); however, this study showed no significant difference among age groups for the presence of symptoms in early-stage breast cancer. Tumor size and nodal status did not differ between age groups, as in the study by Cancello et al. (19).

A study by Walker et al. (20), which has the grouping pattern most similar to the present study, compared immunohistochemical features between three similar groups and showed no significant difference for grading, contrary to the present study. However, hormonal status and Her-2 status were not significantly different in both studies.

The HG was higher in group M, which did not correlate with the current literature (19, 20). However, Bouferraa et al. (21) showed no difference between age groups. Cai et al. (22) assessed factors related to mortality and reported grade as a risk factor, independent of age.

The hormonal and Her-2 status in group Y was similar to that reported by Cancello et al. (19). Sun et al. (23) showed a significantly higher hormone negativity in young patients than in other developing countries.

Although the mean and median Ki-67 levels were >20 in all three groups, group M showed a higher Ki-67 value. The present study differs from the study by Walker et al. (20) in terms of higher Ki-67 levels in group M than in group Y. Moreover, this finding conflicted with those of Kim et al. (6), which might be the reason for the poorer prognosis in group M in addition to the higher grade.

Although luminal-A disease is associated with better prognosis, biological subtyping showed no difference between age groups, similar to the literature (6, 21, 23). Thus, differences in survival between age groups could not be related to only the biological subtype distribution.

Bouferraa et al. (21) presented follow-up data for a median of 96 months, Cancello et al. (19) 68.4 months, Kim et al. (6) 59.9 months, and Cai et al. (22) 43 months. The present study presented follow-up data for a median of 91.6 (52–133) months, which could provide a good perspective to compare survival in young and older patients.

Bouferraa et al. (21) compared patients with nonmetastatic breast cancer with an age cut-off of 40 years. In the present study, the patients were divided into three groups. There was no progression in group Y; however, 11 (13%) patients in group M and two (1%) in group E showed progression (*p* = 0.001). The mean time-to-progression was 52 months in group M and 58 months in group E in the present study, without significant difference (*p* = 0.75). Bouferraa et al. (21) demonstrated higher recurrence in the younger group (28.3% vs. 9.7%, *p* = 0.012), similar to the present study, but longer time-to-recurrence in both groups (95 months for patients age <40 years and 107 months for those aged ≥40 years, *p* = 0.004). Although that study also included stage 3 diseases, contrary to the present study, stage 3 had no difference between age groups in terms of DFS. In both studies, DFS was shorter and recurrence was higher in the younger group. In the present study, the worst prognosis was observed in group M, which could be due to the higher HG and Ki-67 values. Bouferraa et al. (21) showed significant differences in DFS in stage 1 but not in stage 2 disease; however, the present study showed significant differences in both stages (*p* = 0.026 and *p* = 0.024, respectively). Thus, differences in disease stage did not alter DFS according to age in the present study.

Kim et al. (6) assessed patients for 59.9 months a median follow-up period and grouped them according to the 35-year-old cut-off. DFS was 72.8% in the younger group and 86.2% in the older (*p* < 0.001) group. Furthermore, they assessed biological subtypes and found significant differences for DFS in biological subtypes, except for the triple-negative subtype. Additionally, the subtype distribution between age groups in the study by Kim et al. (6) showed a significant difference in contrast to the present study. In addition, Ki-67 positivity (≥20%) was significantly higher (*p* < 0.001) in the younger group (42.1% vs. 29.7%). In the present study, positive Ki-67 values were 50% in groups Y and E and 71% in group M (*p* = 0.013). Additionally, the HG was higher in the young population (54% vs. 42.3%, *p* = 0.002) according to the study by Kim et al. (6), which was higher in group M in the present study. When these two studies are summed up, high Ki-67 levels and HG may explain the worse prognosis in age groups rather than age itself because there were no events in group Y.

Cai et al. (22) also assessed survival by splitting age by decades and reported worse OS in the younger than 40-year-old (youngest group) and elder than 79-year-old groups. The present study compared the three age groups; however, no significant difference was observed in breast cancer-related mortality.

### Limitations

The limitation of this study was the small number of patients aged <35 years.

## Conclusion

This study showed poorer survival in patients aged 35 and 45 years rather than in those aged <35 years. The former group showed higher HG and Ki-67 values. Thus, being very young cannot be considered an independent risk factor for poor prognosis. Rather than age, HG and Ki-67 index are more important determinants for the progression of early-stage breast cancer.

## Data Availability

The raw data supporting the conclusions of this article will be made available by the authors, without undue reservation.
